# Supplementary material for: Inflammatory factors and risk of lung adenocarcinoma: a Mendelian randomization study mediated by blood metabolites
Source: Front Endocrinol (Lausanne). 2024 Aug 27;15:1446863. doi: 10.3389/fendo.2024.1446863 (PMC11384989; doi:10.3389/fendo.2024.1446863)
Supplement: Supplementary file 1 [file DataSheet1.zip › supplementary files/S2/GCST90200413/forest.pdf]

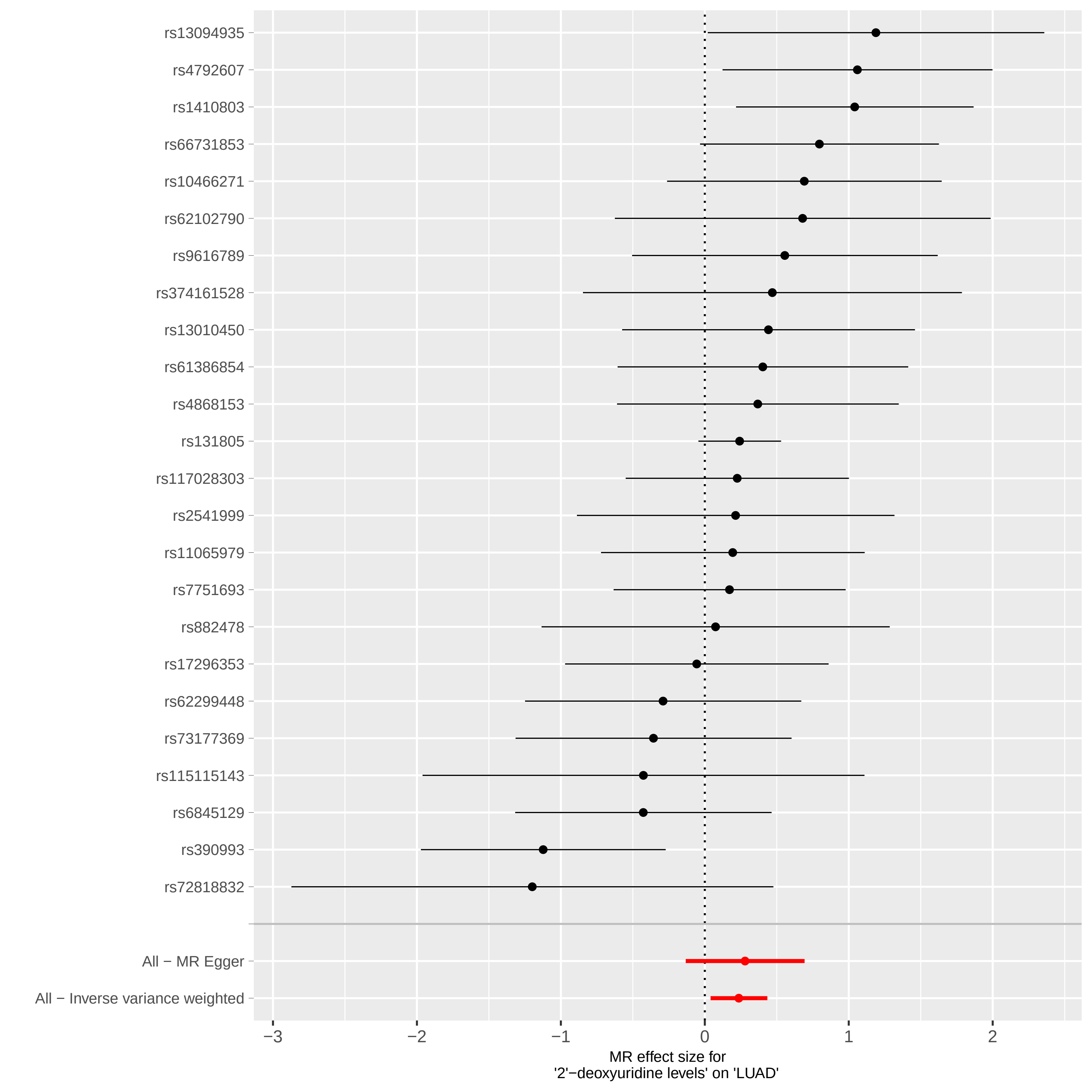
rs13094935
rs4792607
rs1410803
rs66731853
rs10466271
rs62102790
rs9616789
rs374161528
rs13010450
rs61386854
rs4868153
rs131805
rs117028303
rs2541999
rs11065979
rs7751693
rs882478
rs17296353
rs62299448
rs73177369
rs115115143
rs6845129
rs390993
rs72818832
All − MR Egger
All − Inverse variance weighted
−3
−2
−1
0
1
2
MR effect size for
'2'−deoxyuridine levels' on 'LUAD'
